# Innominate truncal and arch blowout with left hemiparesis and right hemothorax followed by delayed cheese-wire perforation of innominate graft

**DOI:** 10.1186/1749-8090-8-109

**Published:** 2013-04-23

**Authors:** Pankaj Kaul, Rodolfo Paniagua

**Affiliations:** 1 Cardiac Surgeon, Leeds General Infirmary, Great George Street, Leeds LS1 3EX, UK; 2 Cardiac Surgical Clinical Fellow, Leeds General Infirmary, Great George Street, Leeds LS1 3EX, UK

**Keywords:** Innominate artery blow-out, Innominate graft perforation, Hemothorax, Hemiparesis

## Abstract

We present the case of a 68 year old Caucasian woman, in extremis, with left hemiparesis and right hemothorax, in hypovolemic shock, secondary to a blow-out of a large penetrating ulcer at the junction of innominate trunk and aortic arch. She underwent interposition graft replacement of innominate trunk and repair of aortic arch, on cardiopulmonary bypass, employing total circulatory arrest and selective antegrade cerebral perfusion and had total resolution of hemiparesis. She, however, represented, 6 months later, with threatened exsanguination after a sternal wire cheese-wired through the sternum and perforated the anteriorly lying innominate graft. Following successful repair, she was found to have an old intramural hematoma of distal arch and descending thoracic aorta and changes suggestive of chronic dissection of the whole of abdominal aorta. This was managed conservatively.

We believe this patient’s presentation initially with a spontaneous innominate blow-out, cardiogenic shock, hemothorax and hemiparesis, and later with cheese-wire perforation of the innominate graft is unique. Her surgical rescue at both presentations was equally unusual, and without surgical precedent to the best of our knowledge. Was the initial innominate blow-out the result of localised innominate dissection, or more unusually, part of retrograde descending thoracic dissection with skip penetration of innominate artery and sparing of the intervening arch? Was it secondary to the minor fall she had sustained 1 week prior to the event, resulting in a false aneurysm or a contained hematoma next to the innominate artery? More intriguingly, did diffuse aortopathy underpin these diverse etiologies and result in penetrating intimal ulcer with blow out in the innominate artery, intramural hematoma in the arch and descending thoracic aorta and dissection in abdominal aorta at different points in time?

We review the current literature for these unusual afflictions of innominate trunk and its origin from the arch of aorta.

## Background

Innominate artery rupture is an extremely rare occurrence in the current era. The usual causes include external or iatrogenic injuries, aneurysmal disease of diverse etiology, malignancy of contiguous structures or aortic dissection. Rupture of an innominate graft by a cheese-wired sternal wire has not been previously reported.

## Case presentation

**Figure 1:**
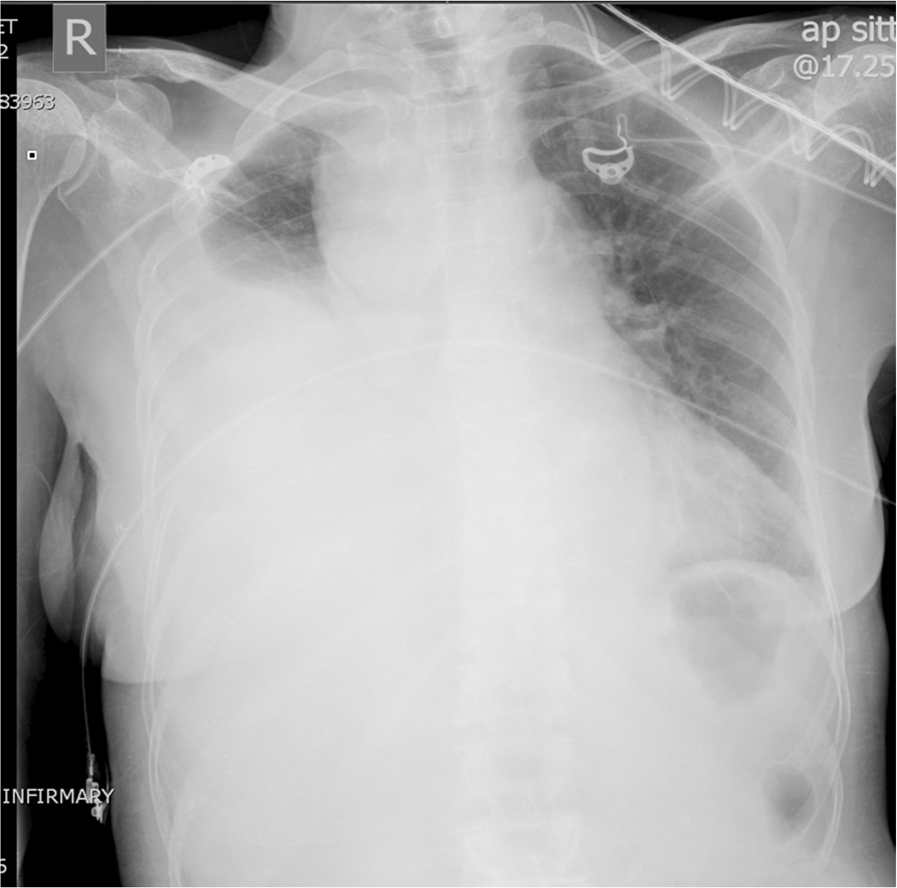
Chest X-ray shows massive right hemothorax, superior mediastinal widening and tracheal deviation to the left.

**Figure 2:**
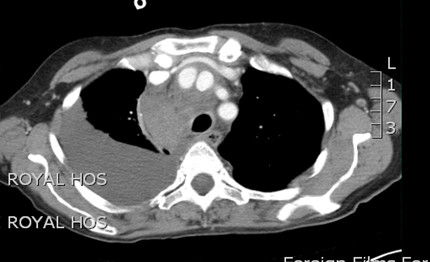
CT scan shows contrast-filled innominate artery false aneurysm next to innominate artery proper, right hemothorax and extensive superior mediastinal hematoma causing tracheal deviation to the left.

**Figure 3:**
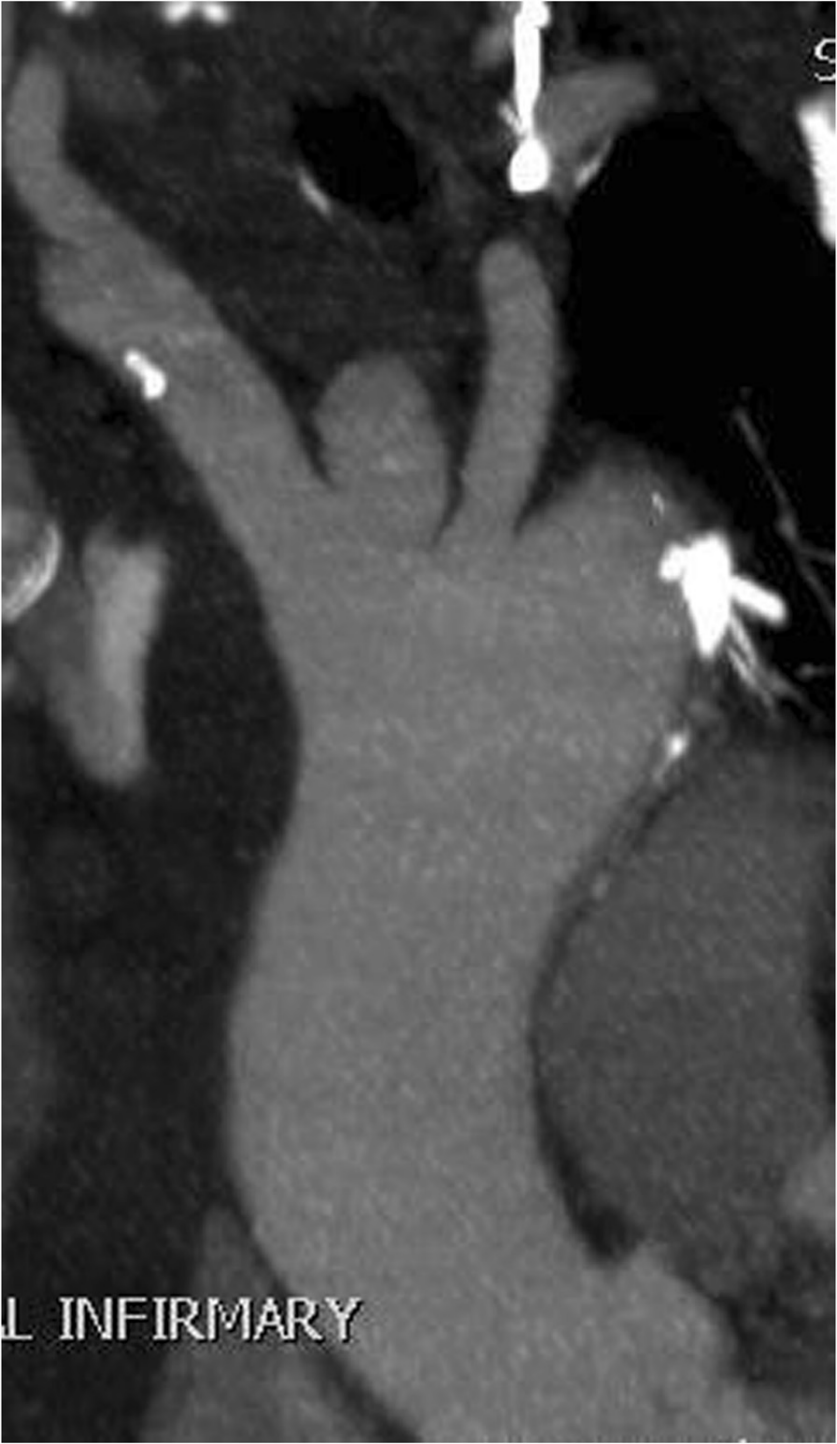
CT scan showing false aneurysm of the innominate artery arising as a blind outpouching at the junction of innominate artery with the arch.

**Figure 4:**
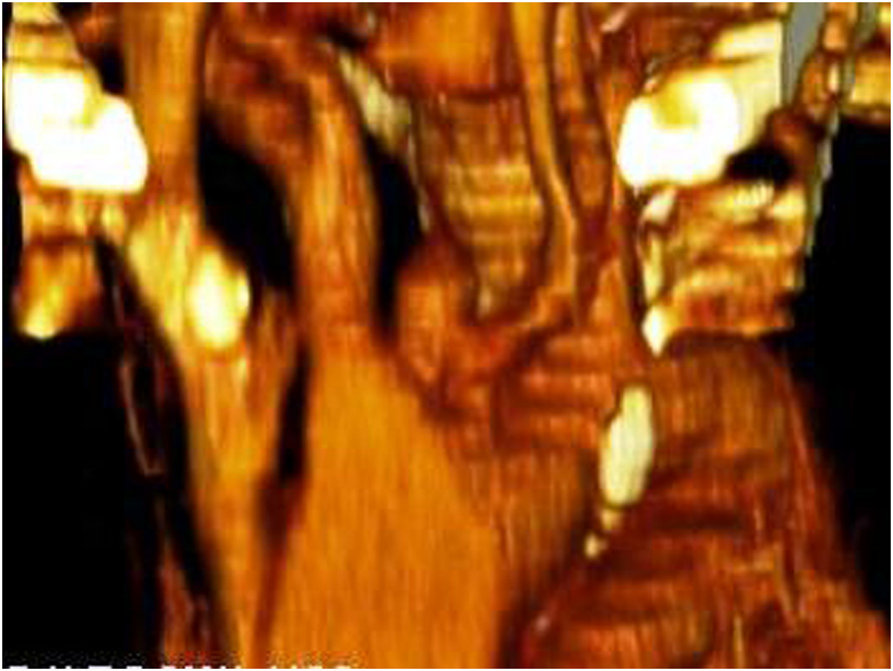
Reformatted picture on CT scan showing false aneurysm of the innominate artery arising as a blind outpouching at the junction of innominate artery with the arch.

A 68 year old woman presented in extremis with 24 hour history of right sided pleuritic chest pain, right CVA with worsening left hemiparesis and respiratory distress. Other significant history included that of hypertension, rheumatoid arthritis, recurrent respiratory infections suggestive of bronchiectasis, right hemi-thyroidectomy for benign disease, new onset Parkinsonism, cholelithiasis and peptic ulcer disease. Intriguingly, she gave a history of fall on the left side with some bruising of leg and gluteal region, 1 week earlier. She was underweight with body mass index of 17.19. Chest X ray showed right hemothorax and superior mediastinal widening (Figure 1). ECG confirmed sinus tachycardia. Contrast CT scan revealed a 3 cm false aneurysm arising from the medial junction of brachiocephalic trunk with the aortic arch, representing a blow out from a medial ulcer at the junction of the brachiocephalic trunk from the aortic arch. There was a large mediastinal hematoma pushing trachea to the left with impending airway obstruction. There was near total collapse of right lung with large right hemothorax. There was no ascending aortic or arch dissection and the left common carotid and left subclavian arteries were normal (Figures 2, 3, 4).

**Figure 5:**
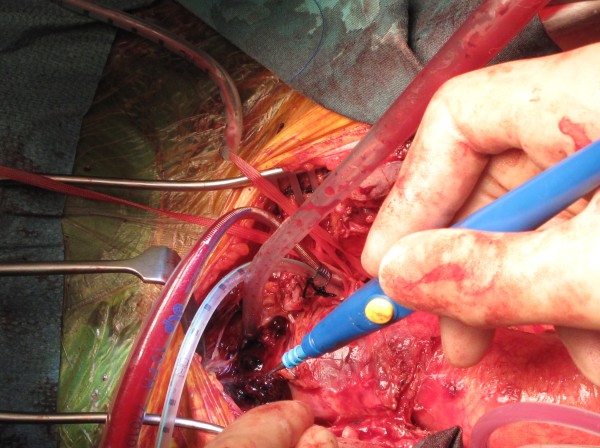
**Intra-operative picture showing fresh clot around the ruptured false aneurysm of the innominate artery.** A thin-walled 10 FR Medtronic cannula has been placed in the left common carotid artery (LCCA) for composite arterial return initially and uni-hemispherical selective cerebral perfusion (SCP) later.

Patient was taken to theatre and right common femoral artery and vein exposed in the groin. Median sternotomy was made. The left common carotid artery (LCCA), the left subclavian artery (LSA), the right subclavian artery (RSA) and the right common carotid artery (RCCA) were taped. Pericardium was opened to reveal fresh blood in the pericardial cavity. There was a 4 cm clot-filled false aneurysm arising from the junction of innominate artery and the aortic arch, occupying a space between the innominate artery and the left common carotid artery, which had ruptured into mediastinum and the right pleural cavity, with more than 1 litre of blood in the right pleural cavity (Figure 5). The pleural cavity was evacuated of blood and clots. The adjoining arch surrounding the take-off of the innominate artery and the proximal half of the innominate artery, beyond the origin of the false aneurysm, were dusky and diseased. Mediastinum surrounding the innominate artery and the adjoining arch was covered with hematoma. The rest of the ascending aorta and root and the arch vessels were normal.

**Figure 6:**
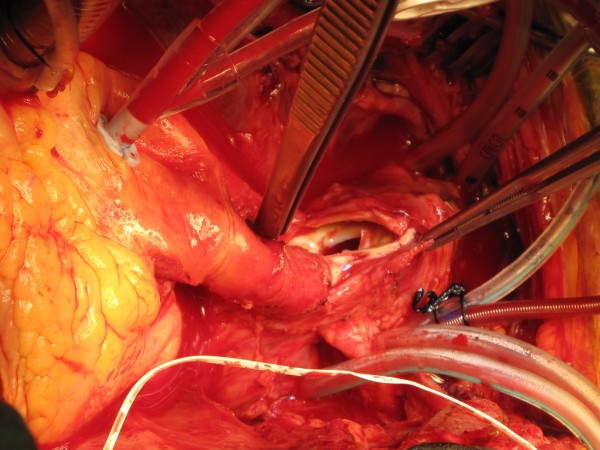
Intra-operative picture demonstrating the false aneurysmal opening at the origin of the innominate artery from the arch.

**Figure 7:**
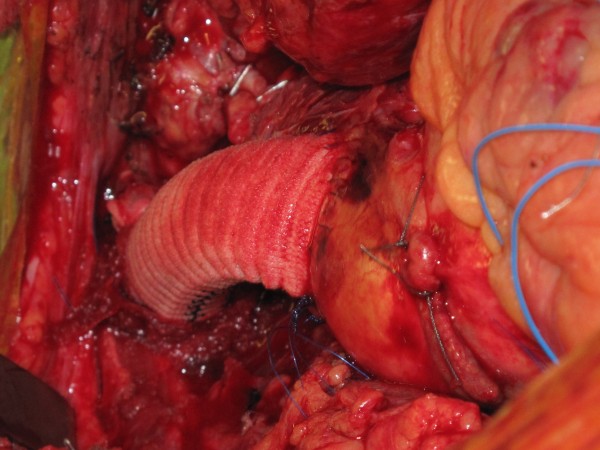
The repair of the aortic arch with bovine pericardial patch is hidden by the 14 mm Vascutek Interposition graft between the ascending aorta and the distal innominate artery.

Cardiopulmonary bypass was established by arterial return into both left common femoral artery and left common carotid artery through a Y connector in the arterial return, and systemic venous drainage by one stage right atrial cannulation. Left ventricle was vented through right superior pulmonary vein vent and patient cooled to 17 C. Aorta was cross clamped and heart arrested with 1 litre of cold blood cardioplegia. At 17 C, the common femoral arterial return was stopped and selective cerebral perfusion (SCP) started through left common carotid artery (LCCA) at 15 ml/kg, aiming at a pressure of 40–50 mm Hg in both radial arteries. The tapes on the RSA, RCCA, LCCA and LSA were snugged down and the aortic cross clamp was released. There was copious return from the distal bifurcation of the innominate artery and the left subclavian artery on temporary release of the tapes and this provided corroborative evidence of the integrity of the circle of Willis along with the fact that pressures in both the radial arteries were equal, around 40 to 50 mm Hg. The origin of the innominate artery from the arch was incised to reveal the mouth of the false aneurysmal opening at the junction of the arch and the innominate artery (Figure 6). The diseased area of the aortic arch surrounding the innominate artery, the entire proximal 4/5^th^ of the innominate artery and the clot filled false aneurysm arising from the arch adjoining the origin of the innominate trunk were excised and sent for histopathology. The resultant defect in the arch was repaired with bovine pericardial patch. The continuity between ascending aorta and the innominate bifurcation was re-established with a 14 mm Vascutex graft (Figure 7). Full body circulation was commenced by reestablishing full femoral flow and unsnugging the tapes on RSA, RCCA, LCCA and LSA. Arterial return was now established through ascending aorta so that the femoral and LCCA cannulae could be removed. Bypass was discontinued, chest closed and patient transferred to ICU in satisfactory condition. Total SCP time was 49 minutes. Histopathology showed features of atherosclerosis in the excised innominate and arch remnants but little else of note.

**Figure 8:**
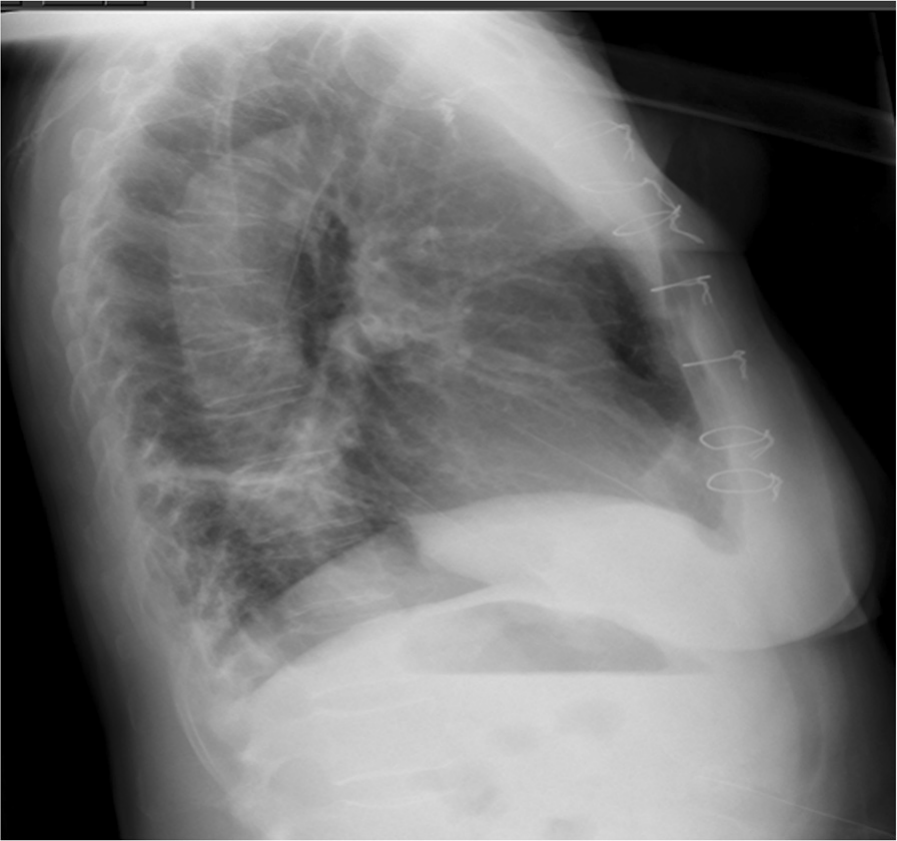
Left lateral chest X-ray showing posterior dislocation of the top sternal wire.

**Figure 9:**
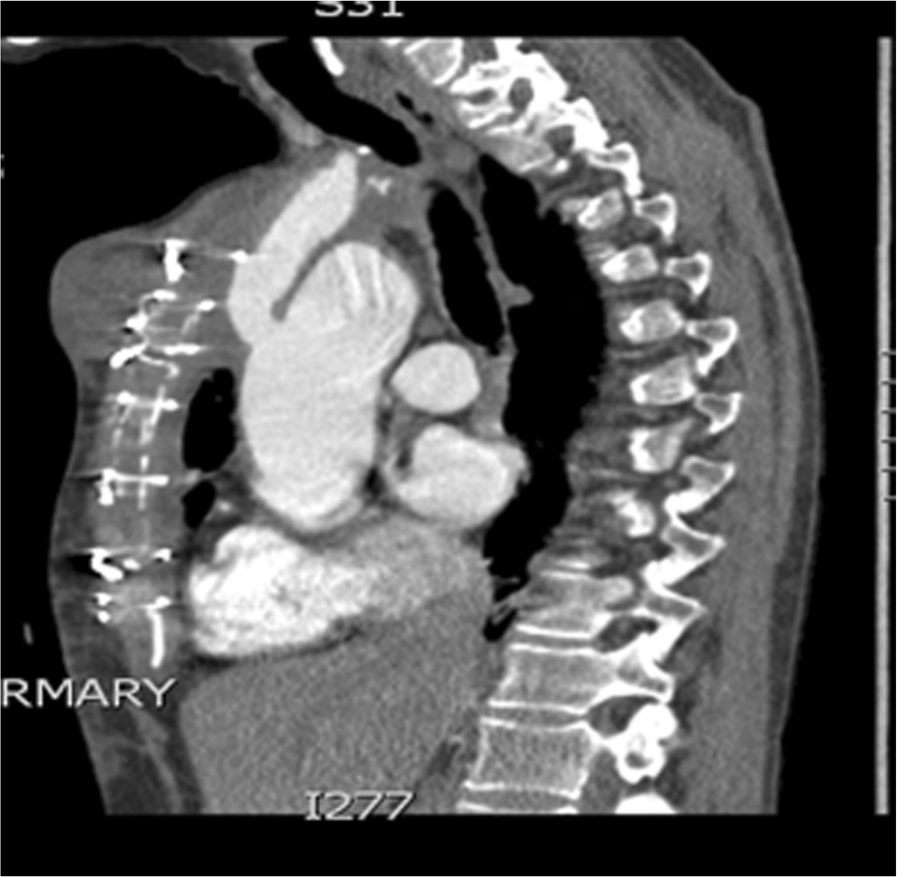
CT scan showing subcutaneous collection overlying manubrium, the top sternal wire with its posterior tip precariously close to the innominate graft, although there is no suggestion of the subcutaneous collection communicating with the innominate graft.

**Figure 10:**
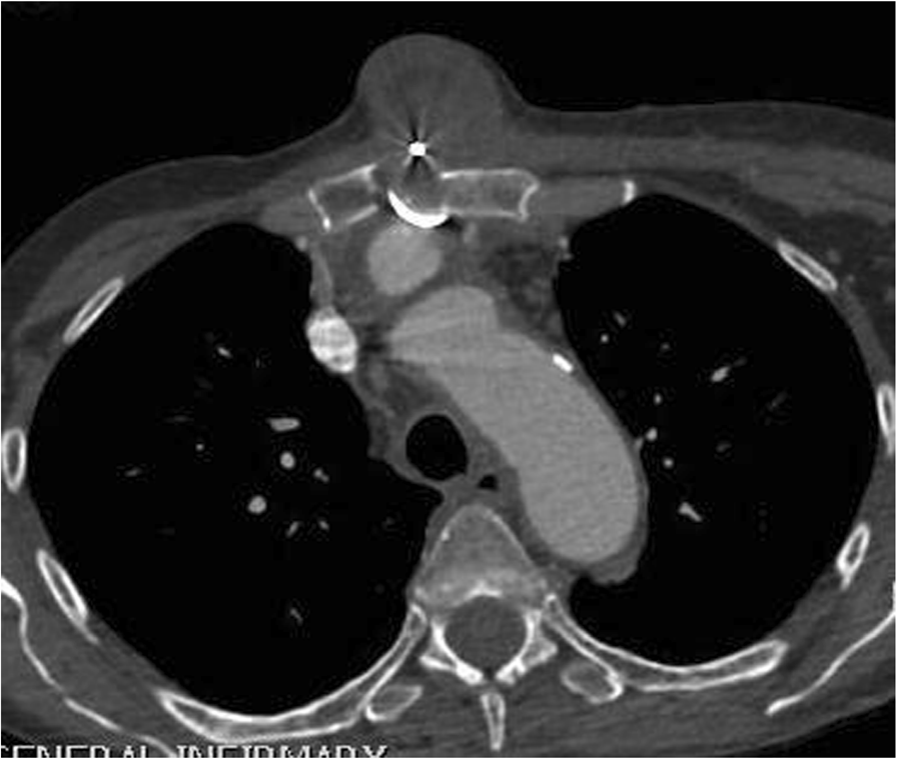
CT scan confirms subcutaneous collection overlying manubrium, manubrial disruption, posterior dislocation of the sternal wire which is seen to lie close to the innominate graft.

**Figure 11:**
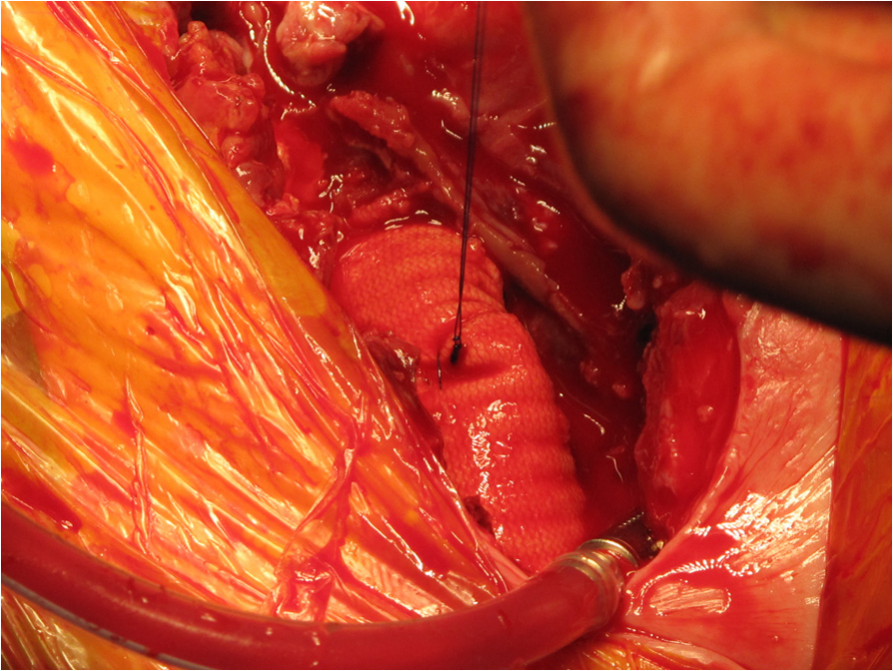
Intra-operative picture showing that the innominate graft perforation by the cheese-wired, posteriorly dislocated sternal wire has been stitched.

In the postoperative period, patient made good cardiac recovery, reasonable neurological recovery, including resolution of left hemiparesis, although swallowing difficulties remained. She was transferred to Stroke Rehabilitation Unit in the district hospital on the 8^th^ postoperative day where she required percutaneous esophagogastrostomy (PEG). She also developed MRSA pneumonia from which she recovered. She was discharged home 15 weeks following initial surgery. However, a month after discharge she represented with a subcutaneous swelling in the upper part of her healed sternal wound. Lateral chest X-ray showed an abnormally dislocated posterior top sternal wire (Figure 8). Contrast CT showed evidence of bilateral lower lobe bronchiectasis. There was a 36 mm non-enhancing subcutaneous hematoma overlying manubrium with no communication with the previous repair. In particular, there was no extravasation of the contrast medium from the innominate graft or the previous arch repair into the subcutaneous collection and no false aneurysm in relation to previous surgery. The top sternal wire had cheese-wired through the sternum and was lying close to the brachiocephalic graft (Figures 9 and 10). The subcutaneous hematoma was evacuated and there was no ongoing bleeding from behind the sternum. However, one week later, following the development of another spontaneous hematoma, she was transferred to theatre. Secondary median sternotomy was made after exposing right femoral artery and vein. The top sternal wire had cut through the sternum and had perforated the innominate graft at the junction of upper 1/3 and lower 2/3, causing a ½ cm perforation in this 14 mm Vascutex interposition graft between the ascending aorta and the innominate bifurcation, which was lying just under the sternum. Femorofemoral bypass was established. Heart was partially dissected away from the sternum, bleeding from the perforation in the innominate Vascutex graft controlled manually till heart and the graft could be completely dissected away from the sternum. The innominate graft was clamped both proximally and distally for 3 minutes and direct suture of the perforation done in two layers (Figure 11). A bovine pericardial patch was incorporated between the sternum and the graft. Routine sternal closure was done and patient transferred to ICU.

**Figure 12:**
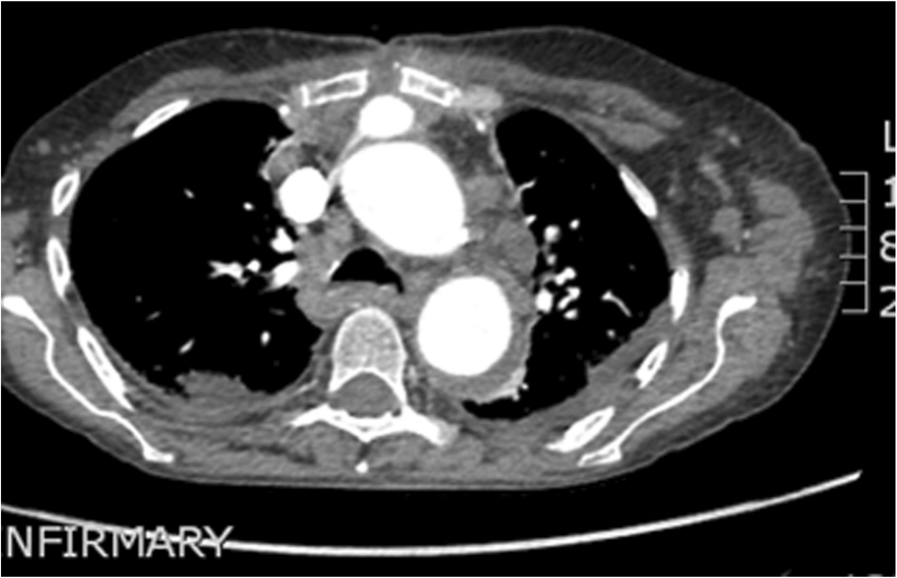
Post-operative CT scan showing old intra-mural hematoma in descending thoracic aorta.

**Figure 13:**
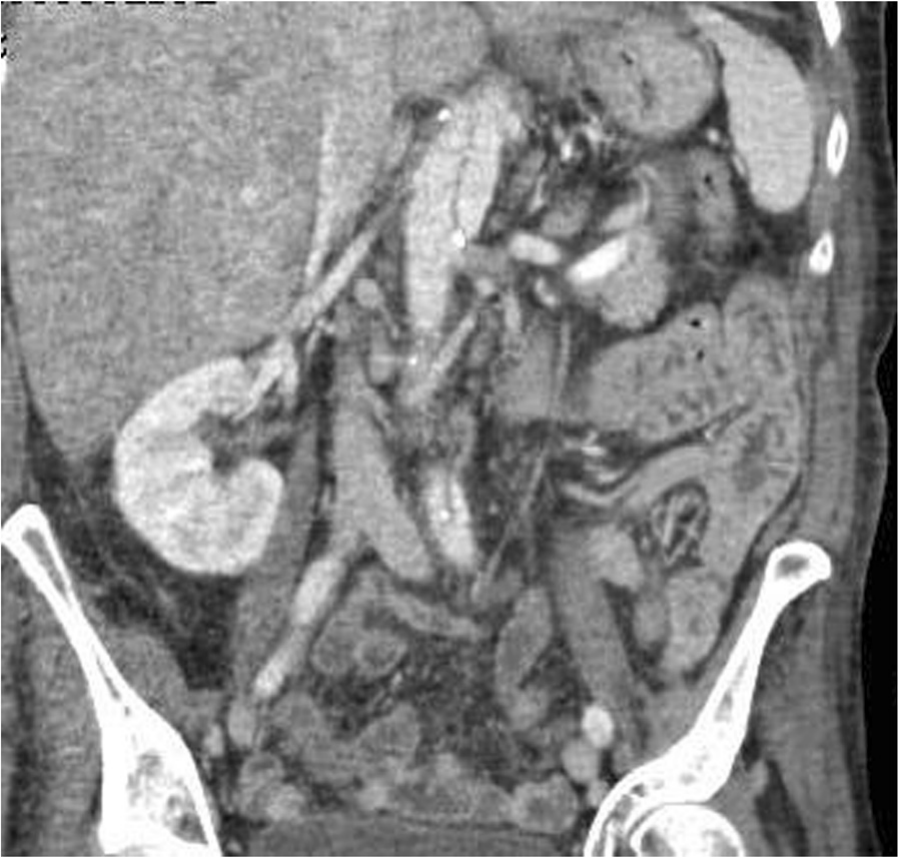
CT scan showing chronic upper abdominal aortic dissection.

Patient made satisfactory recovery but had to stay in hospital for 6 weeks for completion of intravenous antibiotics. A postoperative CT scan to investigate abdominal pain revealed an old intramural hematoma in the distal arch and descending thoracic aorta (Figure 12), with low attenuation on the non-contrast study, extending down into the upper abdominal aorta and changes suggestive of chronic dissection of upper abdominal aorta (Figure 13), with false lumen supplying the celiac axis, left renal artery and IMA all of which demonstrated good opacification. There were ischaemic changes in spleen and left kidney, but none in liver, which showed congestion, and none in bowel. There was cholelithiasis. The aortic dissection recommenced just proximal to the aortic bifurcation and extended into both common iliac arteries with distal extension up to the level of the left common femoral artery. These chronic changes in the thoracic and abdominal aorta were managed conservatively. Patient was transferred to a rehabilitative facility, from where she was transferred home. She has been seen in follow up clinic regularly, last time more than a year following her initial surgery, when she was noted to have made very good progress, maintaining good quality of life.

## Discussion

Innominate trunk can be involved with atherosclerosis, arterites, trauma, dissection and aneurysmal disease of diverse etiology.

Similarly, innominate trunk rupture can follow any of the above disease processes as well as a variety of other conditions. Blunt or deceleration trauma [1,2], operative injury during tracheal surgery [3] or tracheostomy [4], tumor invasion [5], radiotherapy [6], invasive infections [7], tubercular pseudoaneurysms [8], heritable abnormalities of connective tissue like Ehlers- Danlos syndrome [9], bovine aortic arch [10,11], innominate artery aneurysms, caused variously by Takayasu disease, degenerative disease, Syphilis, trauma, Marfan disease and dissection [12] can all result in innominate trunk rupture.

Innominate dissections, with or without innominate rupture, can be spontaneous [13] or secondary to trauma [14,15], heritable connective tissue disorders like Ehlers- Danlos syndrome [16,17], iatrogenic, for example, after axillary perfusion during arch surgery [18], or following aortic dissections [19]. Aortic dissection involves carotid arteries in 28%, celiac, mesenteric or renal arteries in 28%, iliac or femoral arteries in 26%, subclavian arteries in 14%, coronary arteries in 7% and spinal arteries in 1.8% [20]. 5 to 10% patients develop stroke due to innominate/carotid dissection following type A dissection [21]. Iguchi et al reported that among 208 stroke patients presenting within 3 hours of onset, only 2 patients (1%) displayed aortic dissection [22]. Innominate dissection is, however, rarely complicated by rupture of innominate trunk. On the other hand, 30% of patients with ascending aortic dissection died within 24 hours and a further 50% within 48 hours, mostly due to ascending aortic rupture into pericardial or pleural cavities or mediastinum [23]. This difference, in frequency of rupture between aorta and innominate artery, is explained by Laplace’s law as well as the extrapericardial location of the innominate artery, and the support provided by the supraaortic tissues making it resistant to rupture, despite high incidence of innominate involvement in type A dissection [24]. Post traumatic innominate artery rupture has been reported after repair of type A dissection [25], as has been a rupture of innominate artery associated with a localised acute dissection, repaired with a bifurcated prosthetic graft without cerebral complications [26].

Innominate dissection, with or without aortic dissection, may or may not present with neurological symptoms depending on a number of factors including the extent and speed of occlusion and the integrity of circulation through circle of Willis. Rarely, aortic dissections may present as stroke without chest pain or with rapidly deteriorating consciousness [27-29]. A carotid bruit, widened mediastinum on chest X ray, a dissection flap in ascending aorta on echo and a CT chest establish the diagnosis. Innominate or carotid dissection, with or without aortic dissection, is not necessarily a contraindication of surgery. Repair is often followed by amelioration in the degree of disability postoperatively, or less often by complete resolution of symptoms. Alternately, complete resolution after percutaneous stenting of right common carotid artery and innominate artery through right common carotid artery after type A dissection repair, complicated by thrombotic defects in IA and RCCA postoperatively, has been described [30]. Again, bail-out percutaneous external shunt from common femoral artery to right common carotid artery in a patient with type A dissection complicated by innominate dissection and right common carotid occlusion with restoration of normal flow on transcranial doppler in the middle cerebral artery territory has been described, followed by an unsuccessful ascending aortic and hemi-arch replacement [31]. Localised dissection of aortic arch at the origin of innominate and left common carotid arteries with onset of unconsciousness after archery practice in a 43 year old man was managed by an aorto-bicarotid bypass graft on cardiopulmonary bypass with selective cerebral perfusion [32]. Two stage repair of type A dissection extending to innominate trunk and left common carotid artery causing occlusion of RSA and LCCA has been reported. First stage repair involved left sided subclavian-carotid reconstruction followed by ascending aortic, arch and innominate reconstruction [33]. Innominate and carotid dissections not presenting with stroke at initial type A dissection do, however, predispose to delayed neurological problems. In a large study, 42 out of 137 patients with type A dissection following surgical repair had residual innominate dissection, out of which 13 developed focal neurological complications and 6 developed frank strokes at a median follow up time of 3.1 years [34].

Traumatic rupture of innominate artery is more common. In one large series, there were 8 blunt innominate artery disruptions out of 66 blunt aortic and great vessel injuries over a 5 year period. Six were proximal (within 0.5 cm of the origin) and were treated by ascending aortic – distal innominate artery graft with oversewing of the innominate origin or ligation. One each was distal (within 0.5 cm of the bifurcation) and middle and were managed by end to end anastomosis or interposition graft respectively. Cardiopulmonary bypass or aorto-carotid shunting was not utilised in any patient [35]. Surgical approaches to ruptured and unruptured innominate artery aneurysms have been described. In one large series, out of 27 patients with IAA, 3 ruptured, out of which 1 died. 2 high risk patients underwent cervical distal exclusion and cross-over graft. The rest 25 patients had median sternotomy. 10 patients had prosthetic replacement of ascending aorta and/or arch with separate prosthetic graft to the innominate artery. 13 patients had ascending aortic-IA bypass graft with lateral suturing or prosthetic patching of aorta. One patient with infected aneurysm had excision of aneurysm, proximal ligation of IA and transposition of RCCA to LCCA. One patient died [12]. With increasing use of innominate artery for arterial cannulation during surgery of the thoracic aorta [36] and during repair of type A dissection for both arterial return and selective cerebral perfusion to improve neurological outcomes [37], it is possible there might be an increase in iatrogenic innominate artery injuries. However, most cases of supra-aortic aneurysms are asymptomatic and embolization as opposed to rupture represents the greatest risk to the patient. Endovascular repair is an emerging alternative to treatment and is likely to form the mainstay of treatment in future [38].

Our patient presented in extremis with sudden onset of right hemothorax and left hemiparesis and was found to have a rupture of the false aneurysm which originated at the innominate take off from the aortic arch, secondary possibly to an ulcer blow-out. There was extensive mediastinal hematoma around the involved vessels, but, importantly, the rest of the arch and the ascending aorta were normal. In particular there was neither aneurysmal dilatation nor dissection of ascending aorta or aortic root. What was the underlying pathology? Was the rupture traumatic, the false aneurysm secondary to trauma to the innominate take-off at the time of fall 1 week ago, although the trauma at the time did not seem severe? Or was it secondary to a localised dissection that ruptured? Was the dissection secondary to an undiagnosed arteriopathy? The CT scan had suggested a medial ulcer at the junction of innominate artery with the aorta which blew out, initially contained within the false aneurysm, which too ruptured. Six months after the initial episode, CT scan of the aorta showed a dissection of the abdominal aorta upto the iliac bifurcation and hematoma in the descending aorta and distal arch although the CT scan at initial presentation had shown no aortic dissection. Was this an aortopathy not picked up by routine investigations that resulted in innominate and aortic arch weakness resulting in the blow out? Histopathology suggested only a minor degree of atheromatous intimal expansion. Or were the changes of chronic dissection in the descending thoracic and abdominal aorta secondary to the initial operation on the arch? The right hemothorax is explained by the relation of the innominate trunk to the dome of right pleura and the left hemiparesis to either a catastrophic sudden fall in right hemispherical perfusion at the time of the rupture or thromboembolism at a later stage. Involvement of the arch at the mouth of the innominate take off mandated the repair under cardiopulmonary bypass and circulatory arrest with selective cerebral perfusion through the right common carotid artery. Since only that area of the arch which surrounded the innominate take off was involved, only a patch repair of the arch, rather than total replacement, sufficed, along with the interposition graft replacement of the innominate artery.

Our patient’s presentation nearly 6 months after initial surgery with a subcutaneous hematoma without the contrast showing any communication between the graft and the subcutaneous hematoma is easily explained by the fact that the small perforation in the graft caused by the sternal wire that had cut through the sternum and perforated the graft was temporarily sealed by thrombus. Further downward movement of the wire into the graft reopened and extended the perforation with frank bleeding from the wound threatening exsanguination one week later. The innominate graft extended from the ascending aorta, just above the aortic valve, to the innominate bifurcation, on a plane anterior to the ascending aorta, running just behind the sternum in this frail woman with a body mass index of 17.19. There had been very little thymic remnant or pleural fat to sew in front of the graft. Once the offending sternal wire had cut through the sternum, it was only a matter of time before it perforated the anteriorly placed innominate graft. Once proximal and distal control of the graft, above and below the perforation, had been obtained after establishing femorofemoral bypass, the repair of the perforation was easily achieved. A bovine pericardial patch was placed in front of the graft this time to protect it from any further sternal wire migrations.

A postoperative CT scan to investigate abdominal pain revealed an old intramural hematoma in the distal arch and descending thoracic aorta, with low attenuation on the non-contrast study, extending down into the upper abdominal aorta, changes suggestive of chronic dissection of upper abdominal aorta, with false lumen supplying the celiac axis, left renal artery and IMA all of which demonstrated good opacification. The aortic dissection recommenced just proximal to the aortic bifurcation and extended into both common iliac arteries with distal extension up to the level of the left common femoral artery. It was a moot point whether these changes were new, or related to previous surgery, 6 months earlier, which involved proximal bovine pericardial patch repair of the proximal arch at the innominate truncal take-off and the interposition innominate Vascutex graft, employing left femoral – LCCA composite cannulation, or whether this represented an earlier descending thoracic dissection extending antegradely into abdominal aorta and retrogradely to involve the innominate trunk, but somehow skipped the intervening arch, and failed to be picked up on the conventional CT scan. There are some accounts of retrograde extension of aortic dissections, extending from descending thoracic aorta to arch and ascending aorta, but none that are not in continuity [39].

## Conclusions

Spontaneous innominate artery blow-out and rupture is an extremely unusual occurrence in itself and all the usual substrates of causation, like trauma, dissection and connective tissue disorders, tumour or previous surgery, seem to be missing in our case. The 1 week old history of trivial trauma in our patient is probably coincidental and unrelated. Our patient showed no signs of widespread aortic dissection at the time of initial presentation and histopathology did not suggest any obvious underlying localised innominate dissection.

This case report illustrates that a high index of suspicion needs to be reserved for the rare presentation of innominate rupture when left hemiparesis and right hemothrax acutely present together.

Another learning point from this case report relates to the invariable anterior anatomical location of aorto-innominate grafts. This has special relevance to the more commonly constructed trifurcated grafts from the ascending aorta to the innominate, left common carotid and the left subclavian arteries in the debranching procedures preparatory to TEVAR for descending thoracic and arch aneurysms. All such grafts need to be covered with autologous pericardium or an appropriate substitute.

Finally, any presternal swelling in a patient who has had previous surgery involving anterior thoracic aorta should be presumed to have the same origin unless unequivocally proved otherwise.

## Consent

Written informed consent was obtained from the patient for the publication of this report and the accompanying images.

## Abbreviations

CVA: Cerebrovascular accident; IA: Innominate artery; IAA: Innominate artery aneurysm; RSA: Right subclavian artery; RCCA: Right common carotid artery; LCCA: Left common carotid artery; LSA: Left subclavian artery; SCP: Selective cerebral perfusion; MRSA: Methicillin-resistant staphylococcus aureus.

## Competing interests

The author declares that they have no competing interests.

## Authors’ contributions

PK conceived, designed, wrote and coordinated the assembly of the various components of the manuscript. RP revised the manuscript and made crucial intellectual inputs in the design and presentation. Both authors read and approved the final manuscript.

## References

[B1] Dula DJ Hughes HG Majernick T Traumatic disruption of the brachiocephalic artery Ann Emerg Med 1983 12 10 639 641 10.1016/S0196-0644(83)80213-3 6354012

[B2] Chu MY Myers ML Traumatic innominate artery disruption and aortic valve rupture Ann Thorac Surg 2006 82 1095 7 10.1016/j.athoracsur.2006.01.047 16928548

[B3] Deslauriers J Ginsberg RJ Nelems JM Pearson FG Innominate artery rupture. A major complication of tracheal surgery Ann Thorac Surg 1975 20 6 671 7 10.1016/S0003-4975(10)65760-8 1108816

[B4] Ganapragasam A Fatal haemorrhage from the innominate artery complicating tracheostomy J Laryngol Otol 1975 89 8 853 5 10.1017/S0022215100081111 1102622

[B5] Buist LJ Barnes AD Unprecipitated rupture of the brachiocephalic artery during repeated surgery for parathyroid carcinoma Br J Surg 1992 79 11 1158 1467890 10.1002/bjs.1800791115

[B6] Trajbar T Gveric T Kosuta D Ikvosic A Pavic P Zoricic I Adam VN Iatrogenic injury of the brachiocephalic arterial trunk and its branches – case report Acta Med Croatica 2006 60 5 497 9 17217108

[B7] Ichimura H Ishikawa S Hiramatsu Y Sakakibara Y Onizuka M Innominate artery rupture after transcervical drainage for descending necrotising mediastinitis Ann Thorac Surg 2001 71 3 1028 30 10.1016/S0003-4975(00)02439-5 11269423

[B8] O’Leary M Nollet DJ Blomberg DJ Rupture of a tubercular pseudoaneurysm of the innominate artery into the trachea and esophagus. Report of a case and review of the literature Hum Pathol 1977 8 4 458 67 10.1016/S0046-8177(77)80011-7 330386

[B9] Valverde A Tricot JF de Crepy B Bachdach H Djabbari K Innominate artery involvement in type IV Ehlers-Danlos syndrome Ann Vasc Surg 1991 5 1 41 5 10.1007/BF02021776 1997074

[B10] Kaul P Javangula K Ganti S Balaji S Gough M Sivananthan M Lindsay S Continuous selective bilateral antegrade cerebral perfusion through anomalous innominate artery for repair of root, ascending aortic and arch aneurysm – challenges, vagaries and opportunities of bovine arch variant anatomy and review of literature Perfusion 2009 24 2 121 33 10.1177/0267659109106774 19654157

[B11] Roberts CS Sadoff JD White DR Innominate artery rupture distal to anomalous origin of left carotid artery Ann Thorac Surg 2000 69 4 1263 4 10.1016/S0003-4975(99)01426-5 10800837

[B12] Kieffer E Chiche L Koskas F Bahnini A Aneurysms of the innominate artery: surgical treatment of 27 patients J Vasc Surg 2001 34 2 222 8 10.1067/mva.2001.115807 11496272

[B13] Lampropoulos S Theofilogiannakos EK Gkontopoulos A Syncope and cardiovocal syndrome as the result of spontaneous innominate artery dissection J Cardiovasc Med (Haggerstown) 2009 10 10 815 7 10.2459/JCM.0b013e32832d2f31 19571766

[B14] Vadikolias K Helkiopoulos J Serdari A Vadikolia CM Piperidou C Flapping of the dissected intima in a case of traumatic carotid artery dissection in a jackhammer worker J Clin Ultrasound 2009 37/4 221 2 19208421 10.1002/jcu.20556

[B15] Stahmer SA Raps EC Mines DI Carotid and vertebral artery dissections Emerg Med Clin North Am 1997 15/3 677 98 9255140 10.1016/s0733-8627(05)70325-4

[B16] Gdynia HJ Huber R Bilateral internal carotid artery dissections related to pregnancy and childbirth Eur J Med Res 2008 13/5 229 30 18559307

[B17] Mondon K de Toffol B Georgesco G Cassarini JF Ehlers Danlos type IV syndrome presenting with simultaneous dissection of both internal carotid and both vertebral arteries Rev Neurol 2004 160/4 1478 82 10.1016/s0035-3787(04)70934-0 15103277

[B18] Imanaka K Kyo S Tanabe H Ohuchi H Asano H Yokote Y Fatal intraoperative dissection of the innominate artery due to perfusion through the right axillary artery J Thorac Cardiovasc Surgery 2000 69 405 6 10.1067/mtc.2000.107206 10917962

[B19] Zweirzynska E Bec L Sklinda K Walecki J Garlicki M Pniewski J Common carotid artery dissection in the course of acute aortic dissection De Bakey type I Neurol Neurochir Pol 2007 41 472 6 18033647

[B20] Hirst AE Jr Johns VJ Jr Kime SW Jr Dissecting aneurysm of the aorta: a review of 505 cases Medicine (Baltimore) 1958 37 217 13577293 10.1097/00005792-195809000-00003

[B21] Fann JI Smith JA Miller DC Mitchell RS Moore KA Grunkemeier G Surgical management of aortic dissection during 30 year period. Circulation 1995;92:II 113 Neri E, Sani G, Massetti M, Frati G, Buklas D, Tassi R, Giubbolini M, Benvenuti A, Sassi C. Residual dissection of the brachiocephalic arteries: significance management and long term outcome J Thorac Cardiovasc Surg 2004 128 2 303 12 10.1016/j.jtcvs.2004.02.030 15282469

[B22] Iguchi Y Kimura K Sakai K Matsumoto N Aoki J Yamashita S Shibazaki K Hyper-acute stroke patients associated with aortic dissection Internal Medicine 2010 49 6 543 547 10.2169/internalmedicine.49.3026 20228588

[B23] Lindsay J Jr Hurst JW Clinical features and prognosis in dissecting aneurysms of the aorta. A reappraisal Circulation 1967 35 880 8 10.1161/01.CIR.35.5.880 6021777

[B24] Kaul P George R Paniagua R Petsa A Congiu S Innominate truncal dissection and rupture into right pleural cavity following acute type A dissection of aorta with right coronary ostial avulsion and inferior STEMI Perfusion 2012 26 435 40 21565976 10.1177/0267659111408997

[B25] Semrad M Urban T Tosovsky J Kunstyr J A suprasternal false aneurysm caused by posttraumatic innominate artery rupture after dissection type A repair Eur J Cardiothorac Surg 2006 30 6 937 10.1016/j.ejcts.2006.09.010 17070692

[B26] Munakata H Okada K Tanaka H Yamashita T Nakagiri K Okita Y Acute dissection of the innominate artery: a case of report Gen Thorac Cardiovasc Surg 2008 56 3 131 3 10.1007/s11748-007-0205-8 18340513

[B27] Jachinska K Lipczynska- Lojkowska W Kuran W Rozenfield A Grochowska A Szpak GM Lenart J Aortic dissection and carotid artery dissection complicated by ischaemic stroke in a patient with cystic median necrosis Neurolgia i Neurochirurgia Polska 2009 43/6 584 90 20054762

[B28] Morita S Shibata M Nakagawa Y Yamamato I Inokuchi S Painless acute aortic dissection with a left hemiparesis Neurocrit Care 2006 4/3 234 236 16757829 10.1385/NCC:4:3:234

[B29] Su CH Hong TC Chou YS Tsai CH Hou SH Acute aortic dissection presenting as stroke – a case report Acta Cardiologica Sinica 1997 13/3 163 168

[B30] Roseborough GS Murphy KP Barker PB Sussman M Correction of symptomatic cerebral malperfusion due to acute type I dissection by transcarotid stenting of the innominate and carotid arteries J Vasc Surg 2006 44/5 1091 96 17098547 10.1016/j.jvs.2006.05.053

[B31] Schonholz C Ikonomidis JS Hannegan C Mendaro E Bailout percutaneous external shunt to restore carotid flow in a patient with acute type A aortic dissection and carotid exclusion J Endovasc Ther 2008 15/6 639 42 19090632 10.1583/08-2507.1

[B32] Higashi S Yoshida Y Mitsuoka H Dissecting aneurysm at the bases of the brachiocephalic artery and the left common carotid artery due to localised dissection of the aortic arch:report of a case Kyobo Geka 2007 60 7 575 8 17642220

[B33] Belov YV Stepanenko AB Charchian ER Bogopolskaia OM Guleshov VA Surgical treatment of a patient with type 1 aortic dissection and occlusion of brachiocephalic branches by observation Angiol Sosud Khir 2006 12 4 138 43 17679968

[B34] Neri E Sani G Massetti M Frati G Buklas D Tassi R Giubbolini M Benvenuti A Sassi C Residual dissection of the brachiocephalic arteries: significance management and long term outcome J Thorac Cardiovasc Surg 2004 128 2 303 12 10.1016/j.jtcvs.2004.02.030 15282469

[B35] Karmy-Jones R DuBose R King S Traumatic rupture of the innominate artery Eur J Cardiothorac Surg 2003 23 5 782 7 10.1016/S1010-7940(03)00032-0 12754033

[B36] Di Eusanio M Ciano M Labriola G Lionetti G Di Eusanio G Cannulation of the innominate artery during surgery of the thoracic aorta Eur J Cardiothoracic Surg 2007 32 2 270 273 10.1016/j.ejcts.2007.03.050 17553687

[B37] Kaul P How I, do it – Sole innominate cannulation for type A dissection of aorta The Journal of Cardiothoracic Surgery 2012 7 125 10.1186/1749-8090-7-125 PMC3618214 23167966

[B38] Curry M Greenburg RK Morales JP Mohabbat W Hernandez AV Supra-aortic vessels aneurysms:diagnosis and prompt intervention J Vasc Surg 2009 49 1 4 10 10.1016/j.jvs.2008.08.088 19174249

[B39] Kaul P Spontaneous retrograde dissection of ascending aorta from descending thoracic aorta – a case review Perfusion 2011 26 1 215 22 21247986 10.1177/0267659110395804

